# Adaptation of the National Plan for the Prevention and Fight Against Pandemic Influenza to the 2020 COVID-19 Epidemic in France

**DOI:** 10.1017/dmp.2020.82

**Published:** 2020-04-07

**Authors:** Ali Ghanchi

**Affiliations:** Université de Paris. Center of research in Epidemiology and Statistics (CRESS), INSERM, INRA, F-75004, Paris, France and Necker Enfants Malades Hospital. Service d’Obstétrique - Maternité, chirurgie médecine et imagerie fœtales. Assistance publique – Hôpitaux de Paris (AP-HP), Paris, France

**Keywords:** COVID-19, emergency planning, health crisis management, France, pandemic influenza plan, public health emergency of international concern

## Abstract

On Tuesday, March 17, 2020, at noon, France became the third European country to impose a nationwide containment policy in the fight against epidemic coronavirus disease 2019 (COVID-19) viral infection. Announcing that the country was at “war,” President Macron called upon all to play a role in mitigating against further development of contagion. This extreme measure never seen before during peace time was the result of adapting not only the French Pandemic Influenza Plan (PIP) being applied to the national context but also real-time clinical, epidemiological, and scientific information about the evolution of COVID-19 infection in the country. The situation was further complicated by local municipal elections and political agendas by populist opinions. Despite mass communication about the importance of individual behavioral attitudes to counter disease propagation, few heeded government advice. Consequently, the situation rapidly deteriorated with increasing number of cases that started to overwhelm health services. As a result, decisive and immediate action was taken by the State for the national public health interest. This report from the field details the timely events that contributed to this extreme policy decision taken by France. A policy decision that other Western democracies have since applied as the pandemic disseminated across the globe.

The first presidential address about the worsening coronavirus disease 2019 (COVID-19) epidemic in France was received with mixed feelings on Thursday March 12, 2020.^1^ Explaining that the nation was in the “midst of the greatest health crisis never seen before in this century.” Thousands of school children jubilated about nationwide school closures, while families of the elderly were shocked about the confinement of all nursing homes as of immediate effect. It was confirmed at that moment that COVID-19 was restricted to clusters, and infection was contained at phase 2 of the French Pandemic Influenza Plan (PIP).^2^ These harsh measures were to limit further viral dissemination, and the importance of personal hygiene, social distancing methods, and self-confinement wherever possible was strongly advised.

However, only 48 h after the Presidential address (Saturday March 14) with more than 4500 confirmed cases and over 91 recorded fatalities, authorities announced unexpectedly that the country had entered PIP phase 3.^3^ As a result, draconian measures were introduced to further limit social interactions with the coercive closure of all bars, clubs, cinemas, and restaurants. Although paradoxally, local municipal elections due to take place the next day were not canceled, resulting in confusion about the gravity of the situation. Consequently, many were unambivalent about the epidemic, and few heeded guidelines to limit contagion. With more than 5423 reported cases (of which 400 were in intensive care) on Monday March 16, 2020. It was decided by the government (following scientific recommendations) that only nationwide confinement of the whole population would halt the progression of the COVID-19 epidemic in France. Describing that the country was at “war,” President Macron explained that only a concerted national effort would curb disease progression, limit fatalities, and prevent health services from being submerged.^4^ The aim of this report from the field is to outline how the French PIP was adapted to the current COVID-19 epidemic as scientific and epidemiological evidence evolved over time.

France, like other countries, developed their PIP based upon the World Health Organization’s pandemic influenza risk management guidance following the 2009 influenza pandemic, adapting it to their national context.^5^

Updated in October 2011, the current French PIP aims to mitigate against pandemic influenza by reducing casualties to a minimum and preserve the functioning of society (especially economic activities).^2^ It highlights that “uncertainty” is a characteristic of any crisis, advocating that contingency plans need to evolve as the situation unfolds. The PIP consists of the following 4 phases:

- Phase 1: Prevent pandemic influenza from being introduced into the country.
- Phase 2: Restrict viral dissemination and propagation in France.
- Phase 3: Attenuate the effects of an eventual epidemic to a minimum.
- Phase 4: Return to normalcy and business continuity.

As with any contingency plan, preparation is essential. Consequently, the French PIP advocates the need for masks and medical supplies to be stocked in sufficient quantities. Contingencies to ensure the continuity of daily life (birth and death registration, garbage disposal, funeral arrangements, water and sewage treatment, food distribution, electricity production, public law and order, etc.) are also taken into consideration. Furthermore, communication methods are detailed to inform about disease mitigation to dispel misinformation and rumors that create panic.

Cases of COVID-19 were first recorded in Chinese nationals visiting France on January 24 and measures were quickly taken to keep these patients in isolation. Simultaneously contact tracing was carried out to identify other people potentially at risk from infection (French PIP phase 1).^5,6^ As COVID-19 started to spread to other Asian countries, French nationals were repatriated from China as travel restrictions were imposed. Even though they were quarantined, this controversial policy was in line with the French PIP. By February 13, 2020, 11 confirmed symptomatic cases of infection were reported in France, as COVID-19 spread from China to South Korea, Italy, Spain, Germany, and the United States.^6^ While cases in Lombardy started to soar, French authorities downplayed risks to the public, despite calls for border closures by extremist political parties campaigning for election. Government policy advised only self-isolation if possible and confinement of children at home for those returning from Italy. The first French COVID-19 cluster appeared in the Oise region by February 25. Despite protective measures (self-isolation and school closures) applied only to affected towns, clusters started to emerge elsewhere.

As the health system started to be overwhelmed in cluster areas, France entered phase 2 of the PIP on the February 28 with approximately 100 cases of reported infection. The government response was to ban grouping of more than 5000 people thereby canceling all sporting events and concerts. The annual Paris Agricultural salon regrouping farmers from all over France was canceled on the final day to much dismay and confusion. Throughout this time, the importance of handwashing and individual behaviors to limit transmission were constantly droned by authorities. However, public ambivalence may have also been due to the scientific community minimizing the effects of infection comparing it with seasonal influenza. Limited knowledge about COVID-19, advocated that infection was fairly “harmless” with fatalities limited only to high-risk groups, ie, essentially the elderly (a message still communicated until very recently). By March 4, public concern about the epidemic started to result in the first signs of panic resulting in stocks of masks and hydroalcoholic gel to be exhausted.^6^ Consequently, the government requisitioned protective masks, communicating that this form of protective barrier should be reserved only for those who displayed signs of illness and for use by health professionals. In addition to this, the price of hydroalcoholic disinfectant gel was blocked to prevent profiteering. However, at present the stocks of masks and hydroalcoholic disinfectant gel is still precariously low.

Media reporting a thousand infections by March 8, confinement measures, and school closures in whole regions probably amplified a state of panic of the general population. Consequently, people started to stockpile food and supermarket shelves started to empty rapidly.^7^ Despite messages of reassurance from the government that food supply would continue, news coverage of quarantine measures taken in China, Spain, and Italy probably contributed to this behavior in anticipation of a similar response in France, a situation not accounted for by the French PIP because the WHO Pandemic Influenza Risk Management Guide advised against the use of confinement measures.^2,5^

French management of this unprecedented situation has juggled between individual liberties and effective infectious disease control.^8^ The success of nationwide confinement measures observed in other countries to control infection forced France to follow suit. Despite the economic fallout of this approach, declaring “war” on the epidemic has enabled France to use a whole-of-society approach to defeat COVID-19 as quickly as possible. Thereby mobilizing all private and public sectors (eg, the use of industry to produce masks and disinfectant gel). Although experience from the past Ebola epidemic has illustrated that border closures are futile, France lobbied the European Union (EU) to close the Schengen treaty zone to all non-EU citizens to limit viral dissemination. Other European countries have gone 1 step further and closed their borders more for political reasons than infectious disease control.^9^

Significant knowledge gaps about COVID-19 hampered crisis management and PIP implementation. Although the application of the French PIP to a disease that spreads more rapidly than influenza with a higher morbidity and mortality rate is debatable. The virus’s basic reproductive number (Ro) is estimated to be between 2 and 3, although some publications have reported a Ro of 11.^10^ While disease mortality varied according to different populations (3-4% mortality in China, 7% in Italy).^11^ Rising case fatality rates in France as the epidemic progressed and the success of confinement measures experienced other countries contributed to the decision to impose nationwide confinement. Robust mathematical modeling and clinical experience revealed that the infection rate was underestimated, following the same kinetic as the Italian epidemic.^11^ In the absence of mass confinement, approximately 30 million people would be infected with an epidemiological peak reached in 50 days. Clinical observations in France illustrated all age groups over 25 years affected and clinical manifestations ranged from asymptomatic, mild (80% of cases), to severe. Half of intensive care unit (ICU) patients were under 60 years, with 5% requiring respiratory assistance. For unknown reasons, severe complications have also occurred in healthy patients unaffected by extreme age or comorbidities (diabetes, obesity, etc.). It is hypothesized that anti-inflammatory medication may be the root cause for this phenomenon. In contrast children are largely asymptomatic healthy carriers (hence, the closure of schools).

Faced with this novel situation, innovative strategies have been proposed. These have included the use of the military to support the health system. Furthermore, causalities were transported elsewhere in the country to free up space in saturated ICUs. Infringements of confinement measures without a legitimate reason result in hefty fines.

At the time of writing (March 29, 2020), it is too early to say whether these extreme measures have been effective in reducing cases, because of the long incubation period of COVID-19. However, it is only a matter of time before phase 4 is achieved following similar trends in Italy and China.^11,12^ In the aftermath, much will be learned during debrief to improve contingency plans.^13^ Items for debate may include repatriation policy and mass population screening. COVID-19 rapid diagnostic tests have their pros and cons. Unlike influenza, these tests have problems associated with false positives and cost-effectiveness. Furthermore, insufficient human resources available to carry out mass screening in a short time on the entire population should be addressed.

Communication by authorities was clearly inadequate. During the initial stages of the epidemic, there was a clear discordance between the message given by authorities and public perception. Another shortfall is the question of preparedness, with protective personal equipment stockpiles underestimated to deal with an epidemic of this magnitude. Improving disease surveillance also needs to be addressed. In short, debriefings will undoubtedly change the paradigm.
